# Effect of Process Parameters on Joint Performance in Hot Pressure Welding of 6061 Aluminum Alloy to CF/PA66

**DOI:** 10.3390/ma17020329

**Published:** 2024-01-09

**Authors:** Haipeng Zhou, Yang Li, Weidong Liu, Yan Luo, Sansan Ao, Zhen Luo

**Affiliations:** 1 School of Materials Science and Engineering, Tianjin University, Tianjin 300354, China; tjuzhp3868@163.com (H.Z.); ao33@tju.edu.cn (S.A.); lz_tju@163.com (Z.L.); 2 State Key Laboratory of Advanced Welding and Joining, Harbin Institute of Technology, Harbin 150001, China; 3 International Institute for Innovative Design and Intelligent Manufacturing of Tianjin University in Zhejiang, Shaoxing 312000, China; 4 College of Aeronautical Engineering, Civil Aviation University of China, Tianjin 300300, China; 2022012177@cauc.edu.cn

**Keywords:** hot-pressure welding, carbon fiber-reinforced thermoplastic composite, aluminum alloy, process optimization

## Abstract

Polymer–metal hybrid structures combine the merits of polymer and metal materials, making them widely applicable in fields such as aerospace and automotive industries. However, the main challenge lies in achieving efficient and strong connections between the metal and polymer components. This paper uses the jet electrochemical machining (Jet-ECM) method to customize the surface morphologies on 6061 aluminum alloy (AA6061) sheets. The connection between AA6061 and carbon fiber-reinforced PA66 (CF/PA66) is then achieved through hot pressure welding (HPW). The effects of aluminum alloy surface morphology, welding force, and welding time on the mechanical properties and microstructure of the joint are investigated. The optimal process parameters are determined by the design of the experiment. The results show that the aluminum alloy surface morphology has the greatest impact on the mechanical property of the welded joint. The optimal process parameters are surface morphology with wider, shallower, and sparsely distributed grooves on the aluminum alloy surface, the welding force is 720 N, the welding time is 12 s, the welding temperature is 360 °C, the cooling time is 16 s, and the optimal peak load of the joint is 6690 N. Under the optimal parameters, the fracture morphology in the AA6061 side is almost entirely covered with CF/PA66. The joint experiences cohesive failure in most areas and fiber-matrix debonding in a small area.

## 1. Introduction

Optimized lightweight designs often require the use of multi-materials, often with different physical properties, such as different metals [1,2,3] or polymer composites and metals [3,4]. Among different multi-material structures, the metal–carbon fiber-reinforced polymer (CFRP) hybrid structure takes the advantages of both metal and CFRP and has been proven to be superior in terms of fatigue performance, impact properties, and vibration resistance [5]. In recent years, the metal/CFRP hybrid structure has been widely used in the automobile and aerospace industries. For example, the BMW 7 series applied metal/CFRP hybrid components to its various important structures such as the roof beam, B pillar, C pillar, threshold beam, and central channel [6]. Lee et al. [7] demonstrated that a steel/CFRP B-pillar exhibited 10% higher crashworthiness and was 44% lighter compared with the tailor-welded steel B-pillar. Al/CFRP and Ti/CFRP hybrid structures are generally used in wing panels, helicopter blades, fairings, fixed trailing edges, space optical benches, ship hulls, and engine cowlings [8].

**Figure 1:**
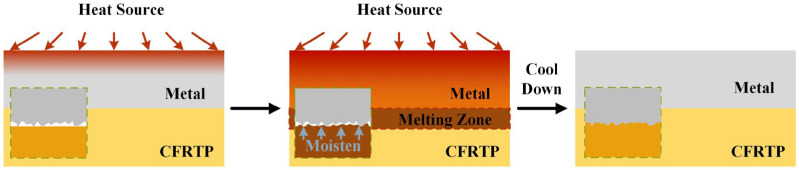
General principle of metal/CFRTP welding.

Since the wide application of metal/CFRP structure, the demand for the joining between metal and CFRP is increasing. However, due to the huge difference in the physical and chemical properties between metal and CFRP, obtaining high-strength metal/CFRP dissimilar joints has become a technical bottleneck. According to the polymer matrix, CFRP can be divided into carbon fiber-reinforced thermoset composite (CFRTS) and carbon fiber-reinforced thermoplastic composite (CFRTP). CFRTS can only be cured one time; therefore, adhesive bonding and mechanical fastening are the main joining methods for it [9,10]. In comparison with CFRTS, CFRTP has weldability, and welding is considered a promising method for joining CFRTP and metal. The general principle of metal and CFRTP welding is using a certain heat source to heat the metal workpiece to a temperature that is above the melting point of the CFRTP. Heat is transferred to the metal/CFRTP interface through heat conduction of the metal, and the CFRTP melts and moistens the metal surface. After the welding is completed, the joint cools to room temperature, and a metal/CFRTP joint forms. Figure 1 shows the general process of metal/CFRTP welding.

The main welding methods of metal/CFRTP include laser welding [11,12,13,14,15,16,17,18], friction welding [19,20,21,22,23,24,25,26,27], ultrasonic welding [28,29,30,31,32,33,34], hot pressure welding [35,36,37,38,39,40,41], and so on. Among them, hot pressure welding (HPW) has the characteristics of simple equipment structure, environmental friendliness, ease of automation, etc., and has a broad development space and application prospect. A number of scholars have conducted studies on the HPW of metal to CFRTP. Barrak et al. [35] prefabricated two holes on the aluminum alloy sample and used HPW to connect the hybrid structure of aluminum alloy and polyamide. The maximum tensile–shear strength of the joint was 2.5 MPa. Zou et al. [36] used hydrofluoric acid (HF) to etch Ti-6Al-4V alloy (TC4) to obtain low roughness surface and used HPW to connect TC4 and poly(ethylene terephthalate) (PET). This study showed that roughness is not a factor in determining joint strength. The factor that can improve the interfacial bonding of metals and polymers is the synergistic effect of surface particle anchoring and chemical bonding. Du et al. [37] used an anodizing method to prepare microporous structures on the surface of aluminum alloy and used ultrasonic-assisted hot pressing technology to weld polypropylene/aluminum alloy dissimilar materials. Liu et al. [38] investigated the effect of laser texturing on the joint performance in HPW of TC4 titanium alloy and CFRTP. The results showed that the textured TC4 surface improved the wettability of molten CFRTP to TC4, thereby enhancing the tensile–shear force of the joint. Saborowski et al. [39] used an Nd/YVO_4_ nanosecond laser system to create a pin structure with scalable height through single-pulse drilling on 6082 aluminum alloy and connected it to polyamide 6 using HPW. The experimental results showed that pulse drilling pin structures show excellent wetting behavior.

At present, the commonly used method to improve the strength of metal/CFRTP hot-pressure welded joints is to increase the macro- or micro-mechanical interlocking between both. The macro-mechanical interlocking is mainly conducted by prefabricating holes [35], raised structures [38,39], or combined use [40] on the metal surface. During the HPW process, the molten CFRTP matrix will flow into the holes, or the raised structures will be inserted into the CFRTP matrix and form an interlocking structure after cooling. The micro-mechanical interlocking is fulfilled by creating microporous structures [37] or groove structures [41] on the metal surface. One difference between the two structures is that the microporous structure is highly random, while the groove structure can be customized and, therefore, has better controllability. For example, Liu et al. [38] investigated the influence of textured grid width on the tensile–shear force of the TC4/CFRP joint made by HPW. The experimental results indicated that laser texturing obviously improved the TC4 surface roughness and wettability of molten CFRP, which increased the interfacial joining area and thus enhanced the shear force of the joint. Zhang et al. [41] investigated the effect of a laser-textured surface on the HPW of 6061 aluminum alloy to glass fiber-reinforced thermoplastic (GFRTP). The results showed that the higher the surface roughness of aluminum alloy, the better the wettability of molten GFRTP and the stronger the joint mechanical properties. Liu et al. [42] studied the effect of groove width and groove depth on the joint strength of Al/CFRTP. The results show that the tensile–shear strength of CFRTP/Al joints first increases and then decreases with the increase in groove width and groove depth. Rodríguez-Vidal et al. [43] investigated the effect of textured groove structure density, structure depth, and cavity angle on the laser-welded steel/PA6-GF30 joints. They found that the textured groove structure density was the primary influencing factor for the joint strength, while the structure depth and cavity angle had little effect. Liang et al. [44] investigated the effect of texturing direction (0°, 45°, 90°) on the Ti/GFRTP laser welded joint strength. The tensile–shear strength could reach the maximum when the texturing direction was perpendicular to the tensile direction (0° texturing). However, these studies did not control the total surface areas of the textures to be equivalent, so it is difficult to compare which structure is better.

This study takes 6061 aluminum alloy and carbon fiber reinforced nylon 66 composite material (CF/PA66) as the research materials. Jet electrochemical machining (JET-ECM) was used to customize the groove size and quantity on the aluminum alloy surface. To better compare the effect of groove size on joint performance, this paper created four surface morphologies under the premise that the textured surface areas are equivalent. Based on a customized HPW machine, the effects of metal surface morphology, welding temperature, welding time, and welding force on the joint performance were investigated.

## 2. Experimental Procedure

The experimental materials, methods, and equipment will be introduced in the following part.

### 2.1. Experimental Material

In this study, 6061 aluminum alloy (AA6061) and CF/PA66 were used as the experimental materials. The dimensions of the aluminum alloy and CF/PA66 sheets were 100 mm × 40 mm × 1.5 mm^3^ and 100 mm × 40 mm × 3 mm^3^, respectively. Table 1 gives the chemical composition of 6061 aluminum alloy. The CF/PA66 sheets were injection molded by pellets provided by EMS (Grivory^®^GCL-4H, Suzhou, China). The physical properties of the CF/PA66 are listed in Table 2. Since the CF/PA66 material is hygroscopic, the CF/PA66 sheets were dried at 80 °C for 4 h before welding.

**Figure 2:**
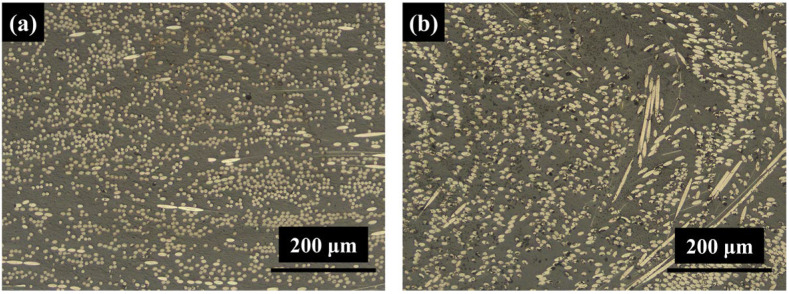
Microscopic morphologies of CF/PA66 sheet: (**a**) cross-section; (**b**) longitudinal section.

The microstructure of the cross-section (perpendicular to the injection direction) and longitudinal section (parallel to the injection direction) of the CF/PA66 (taken by an optical microscope, Zeiss Axio VerT.A1 produced by Carl Zeiss Suzhou Co., Ltd., Suzhou, China) is shown in Figure 2. The gray part is the polymer matrix, and the white part is the carbon fiber. It can be seen that most of the fibers are round in the cross-section and oval in the longitudinal. This is because the fibers will flow along the injection direction.

### 2.2. Experimental Methods

#### 2.2.1. Aluminum Alloy Surface Treatment

**Figure 3:**
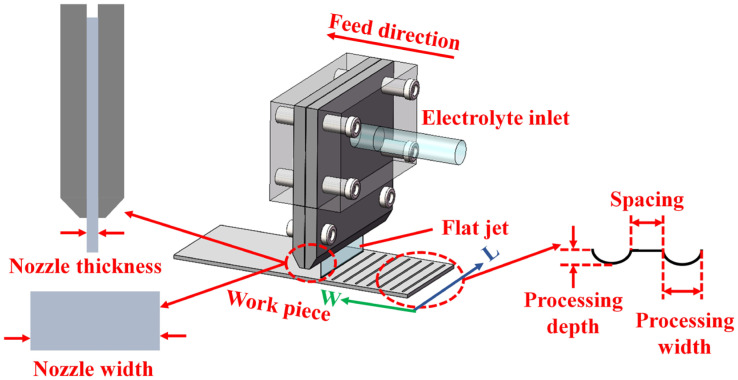
Schematic diagram of the Jet-ECM process.

**Figure 4:**
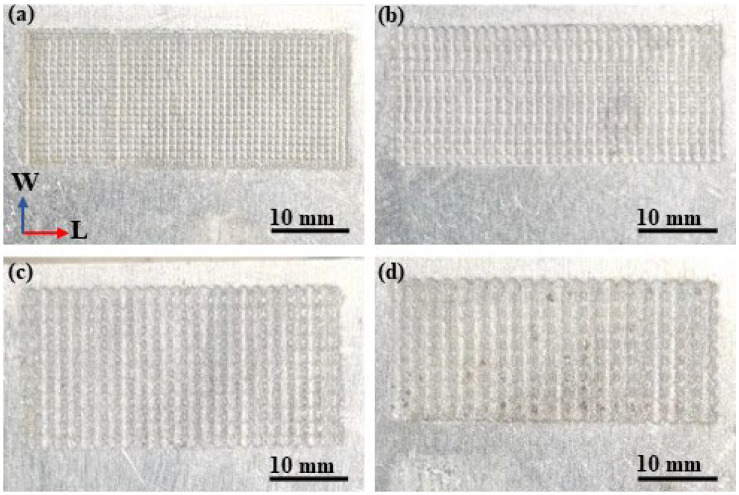
Macroscopic view of the four surface morphologies of aluminum alloy sheet: (**a**) No. 1; (**b**) No. 2; (**c**) No. 3; (**d**) No. 4.

The aluminum alloy surface was textured using jet electrochemical machining (Jet-ECM) technology (Civil Aviation University of China, Tianjin, China), which removes material using the principle of anodic oxidation dissolution by spraying electrolyte from a cathode nozzle onto the anode workpiece. The schematic diagram of the processing process is shown in Figure 3. The workpiece and tool were fixed on a self-developed machining platform, and the electrolyte was passed through a rectangle nozzle. After the groove processing in one direction was completed, the workpiece was rotated 90° and then processed the grooves in the other direction. The designed dimensions of the grooves are given in Table 3. The design principle of the four surface morphologies is to keep the total surface area of grooves approximately equal. According to the designed dimensions of the grooves, a trial-and-error method was used to determine the Jet-ECM processing parameters, as shown in Table 4. Figure 4 (taken by a mobile phone camera) shows the macroscopic view of the four surface morphologies of the processed aluminum alloy workpieces. Among them, the No. 1 surface morphology has the narrowest, deepest grooves and the largest quantity of grooves, and the No. 4 surface morphology has the widest, shallowest, and lowest quantity of grooves.

#### 2.2.2. Hot Pressure Welding

**Figure 5:**
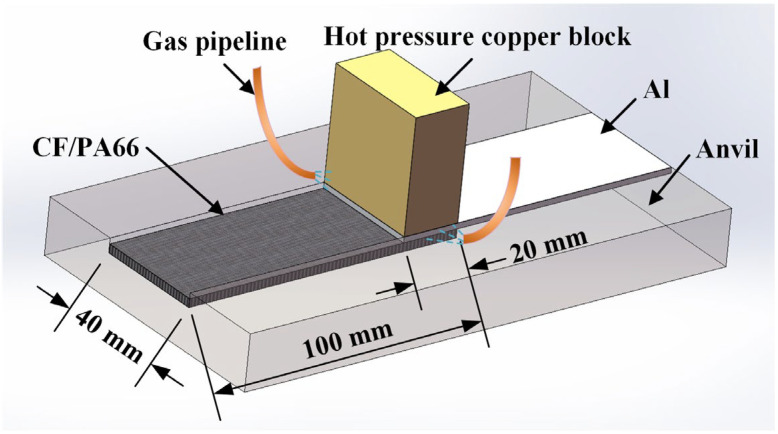
Schematic of the welding setup.

Figure 5 shows the schematic of the HPW setup. Two workpieces were placed in a lap-shear configuration, where a 6061 aluminum alloy sheet was placed on top of a CF/PA66 sheet. The size of the lap area was 20 mm × 40 mm. A copper block with size of 20 mm × 40 mm was used to heat up the aluminum sheet and apply welding force to the materials system. Two air-blowing pipes were distributed on both sides of the copper block. After the welding is completed, air is blown to the welding position to cool the weldment rapidly. After the air blowing was completed, the copper block was raised, and the welding was completed.

Based on the preliminary tests, a four-level orthogonal array test with five factors was designed, as shown in Table 5. Five samples were welded for each parameter, where four samples were used for tensile–shear test, and one sample was used for cross-sectional microscopy. After getting the tensile–shear test results, an online software, SPSS26.0 (Statistical Product and Service Software), was used to analyze the tensile–shear data.

According to the orthogonal array test results, the three parameters (surface morphology, welding force, welding time) that have the greatest influence on the tensile shear property were determined, and univariate tests were carried out. The experimental parameters are shown in Table 6.

**Figure 6:**
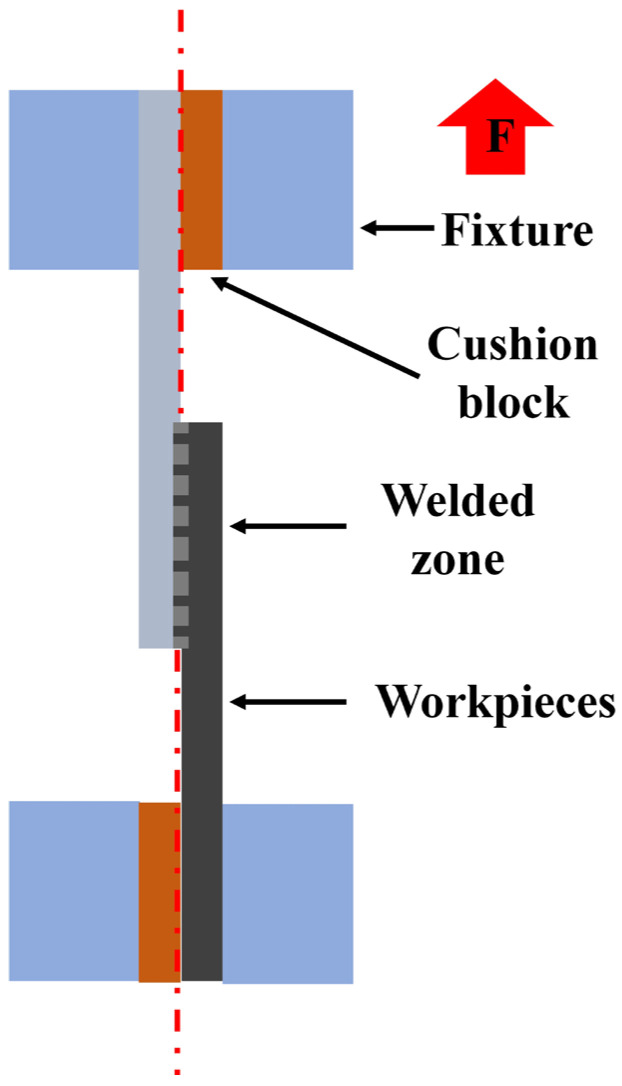
Schematic diagram of tensile–shear test.

Tensile–shear tests were conducted on a microcomputer-controlled electronic universal testing machine with 100 kN capacity (produced by Shenzhen Wance Testing Machine Co., Ltd., Shenzhen, China). The cross-head speed was 2 mm/min. During the test, two pads with the same thickness as the workpieces were added to both ends of the workpieces to ensure that the workpiece is subject to upward tensile force along the same straight line, as shown in Figure 6.

Standard metallography specimen preparation method was used to reveal the cross-section morphology of the joint. The surface of the metallographic specimens was polished with sandpaper of different grits from 200# to 2000#. A PG-1A metallographic polishing machine was used for polishing. The polished specimens were cleaned with alcohol. The cross-section of the joints was observed by an optical microscope (Zeiss Axio VerT.A1 produced by Carl Zeiss Suzhou Co., Ltd., Suzhou, China). Energy spectrum analysis (EDS) was used to analyze the chemical elements on the metal/CFRTP interface. The joints’ fracture morphologies after tensile–shear test were observed by a tungsten filament scanning electron microscope SU1510 (Hitachi High-Technologies Corporation, Tokyo, Japan). Due to the poor electrical conductivity of the CF/PA66, a low vacuum coating instrument (Leica EM ACE200, Leica Microsystems Limited, Hong Kong, China) was used to spray gold on the composite surface before conducting SEM observation, and the gold spraying time was set to 50 s.

## 3. Results and Discussion

### 3.1. Orthogonal Experimental Results

Table 7 shows the tensile–shear test results of the orthogonal array test. To explore the degree of effect of each factor on the joint tensile–shear property and determine the optimal level and combination of the process parameters, the orthogonal array test is analyzed by range analyses. The range analysis has the advantages of easy calculation, simple analysis, and intuitionistic data, which are always used in the theoretical analysis of experimental results.

Table 8 shows the range analysis of the orthogonal array test, where K*_i_* is the sum of all peak loads at level *i* of each factor. For example, K_1_ represents the sum of all peak loads of experiments with a temperature of 320 °C. According to K*_i_*, the optimal level and combination can be obtained. R is the range (max(K_1_, K_2_, K_3_, K_4_)−min(K_1_, K_2_, K_3_, K_4_)) that reflects the degree of effect of each factor and level on the tensile–shear force. The larger the R value, the greater the degree of effect of this factor on the peak load. It shows that the surface morphology has the greatest influence on the joint strength, followed by welding force, welding time, welding temperature, and cooling time. The optimal parameters were No. 4 surface morphology, welding force of 720 N, welding time of 12 s, welding temperature of 360 °C, and cooling time of 16 s.

**Figure 7:**
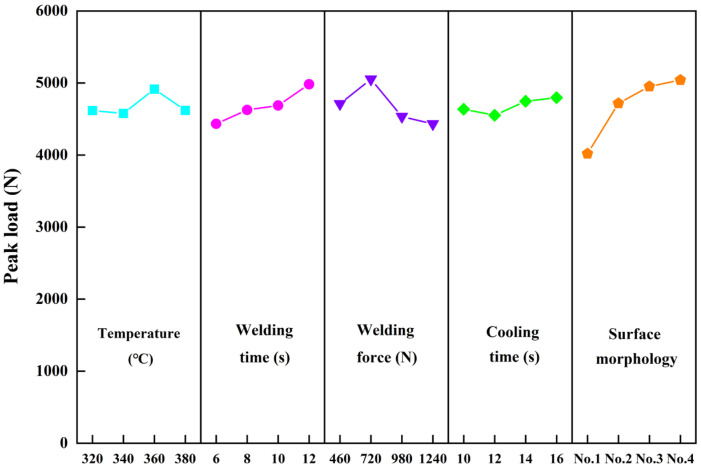
The main effect plot of process parameters on the peak load in tensile–shear test.

A more intuitive display of the effect of each factor is shown in Figure 7. The most important three factors (surface morphology, welding force, and welding time) will be discussed in Section 3.2. The cooling time has a limited influence on the peak load, indicating that 10 s was enough for the joint to cool down. It is noted that temperature also has limited influence in this study. A possible reason is that the welding temperature in this study refers to the temperature of the heated copper block, not the temperature of the welding interface. Therefore, no matter what welding temperature was used, the connection started when the interface temperature reached the melting point of the CF/PA66. Our next work will use more accurate test methods to study the impact of the welding interface temperature on joint performance.

**Figure 8:**
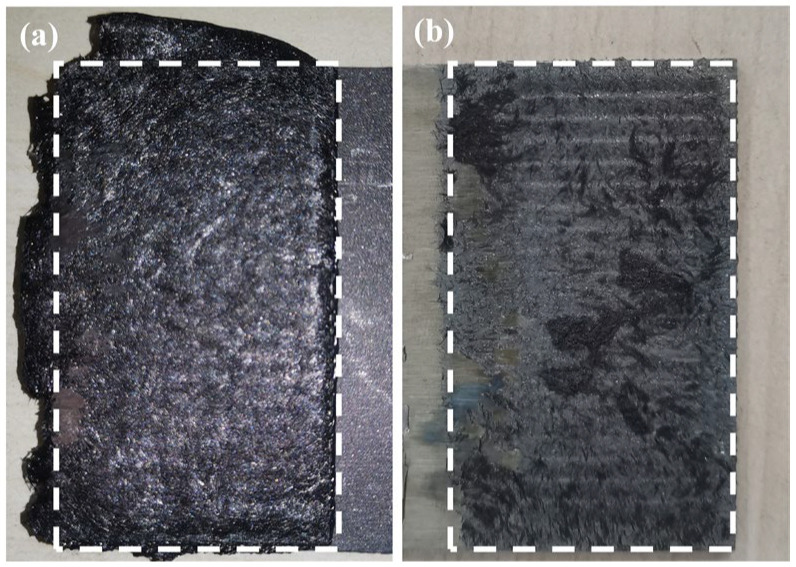
Fracture morphologies: (**a**) CF/PA66; (**b**) AA6061.

Figure 8 shows the fracture morphologies under the optimal parameters. It can be seen that the surface of AA6061 was entirely covered by the CF/PA66. More details about the fracture behavior will be discussed in Section 3.2.4.

**Figure 9:**
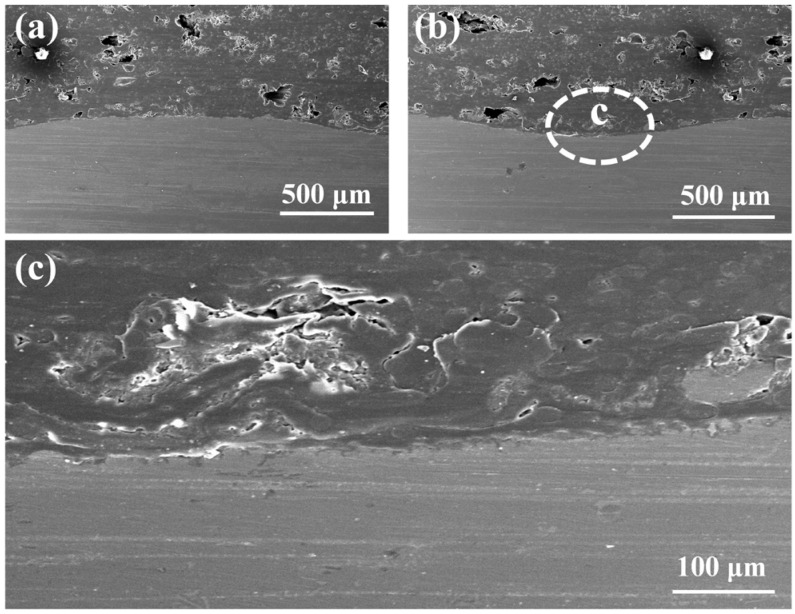
Cross-sectional morphologies of a joint made with the optimal parameters: (**a**) the convex part of the cross-section; (**b**) the concave part of the cross-section; (**c**) the magnification of the area c in (**b**).

Figure 9 shows the SEM cross-sectional morphology of a joint made by the optimal parameters. A tight bonding between the AA6061 and CF/PA66 can be observed at the interface. Some mechanical interlocking between the CF/PA66 and AA6061 can be seen in the magnified view, as shown in Figure 9c. Note that some tiny pores can be observed in the interior of the CF/PA66. These pores do not appear at the interface, so it is speculated that these pores originally exist within the polymer matrix.

### 3.2. The Influence of Process Parameters on the Joint Strength

#### 3.2.1. The Influence of Surface Morphology

**Figure 10:**
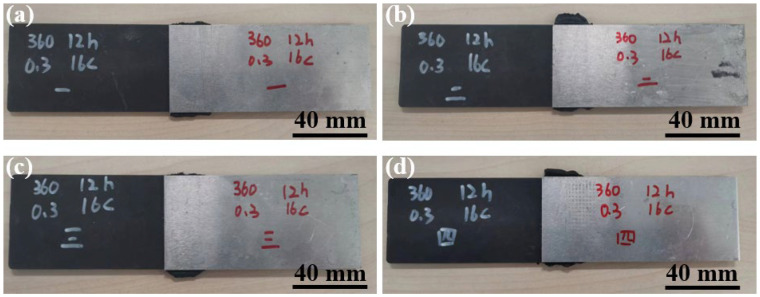
Macroscopic morphologies of Al/CFRTP joints: (**a**) No. 1; (**b**) No. 2; (**c**) No. 3; (**d**) No. 4.

Figure 10 shows the macroscopic morphologies of joints made with different surface morphologies. No obvious difference can be observed among the four joints.

**Figure 11:**
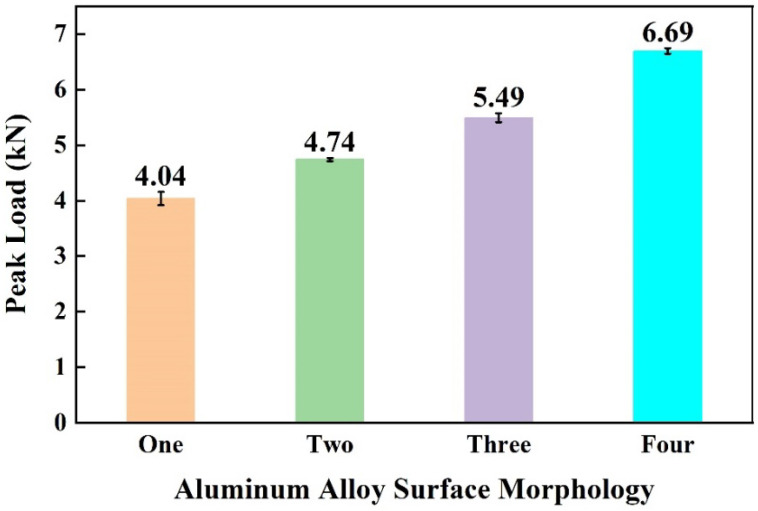
Effect of aluminum alloy surface morphology on the peak load of joints.

**Figure 12:**
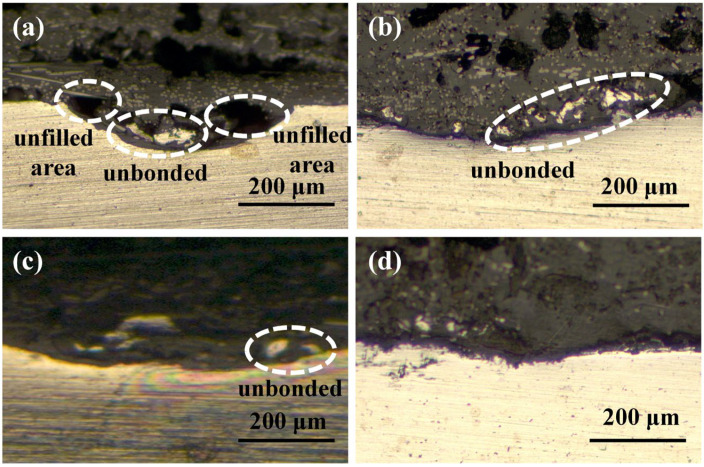
Cross-sectional morphologies of joints made with different aluminum alloy surface morphologies: (**a**) No. 1; (**b**) No. 2; (**c**) No. 3; (**d**) No. 4.

Figure 11 shows the peak load of joints made with different aluminum alloy morphologies. With a comparable total surface area of grooves, the aluminum surface had a wider, shallower, and smaller quantity of grooves, resulting in stronger joints. From the cross-section of the joint, the surface with a narrow, deeper, and larger quantity of grooves will result in an unfilled or unbonded area, as shown in Figure 12a–c. One possible reason is that when two surfaces with comparable surface areas are subjected to the same force, the surface with more densely arranged grooves will disperse the force applied to the grooves, reducing the force that promotes the flow of molten CFPA66 into the grooves.

#### 3.2.2. Effect of Welding Force

**Figure 13:**
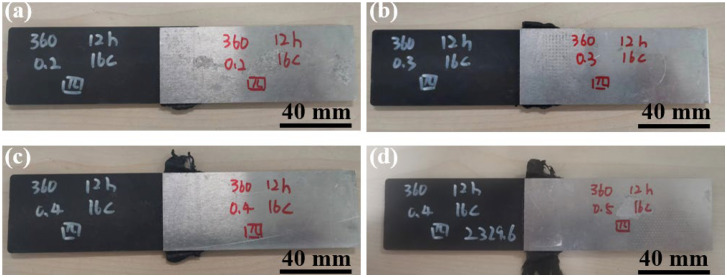
Macroscopic morphologies of Al/CFRTP joints under four welding forces: (**a**) 460 N; (**b**) 720 N; (**c**) 720 N; (**d**) 1240 N.

Figure 13 shows the joints made with different surface morphologies welding forces. As the welding force increased, the extrusion amount of CF/PA66 at the edge showed an increasing trend.

**Figure 14:**
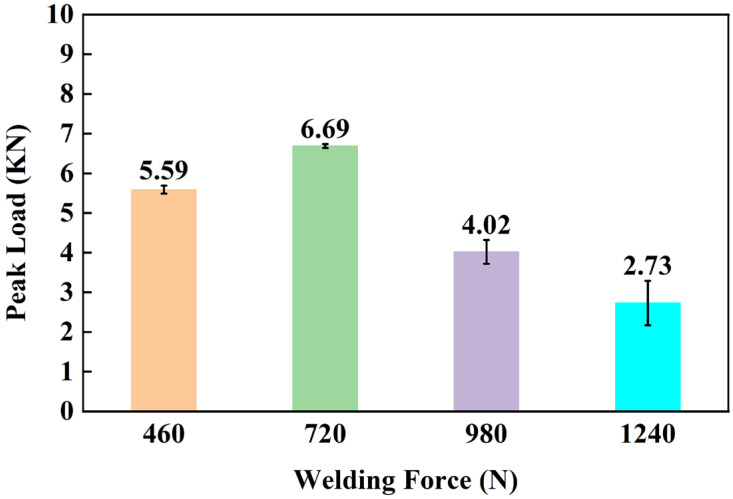
Effect of welding force on the peak load of joints.

**Figure 15:**
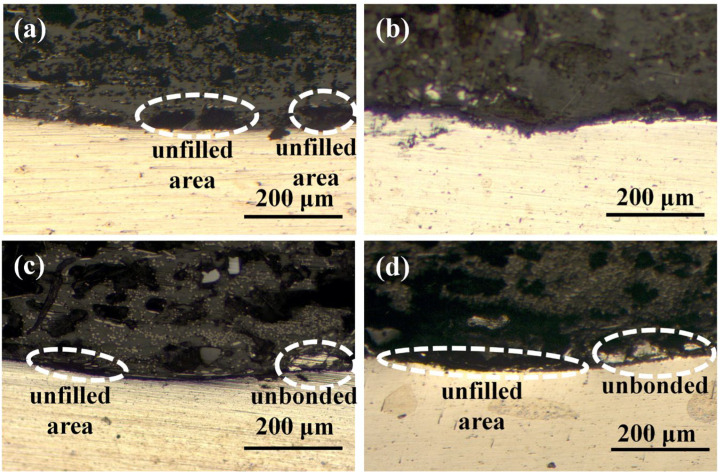
Cross-sectional morphologies of joints made with different welding forces: (**a**) 460 N; (**b**) 720 N; (**c**) 980 N; (**d**) 1260 N.

Figure 14 shows the effect of welding force on the peak load of joints. The peak load of joints increased first and then decreased with the increase in welding force. This can be explained by the cross-sectional microstructures of Al/CFRTP joints, as shown in Figure 15. When the welding force was relatively low, the grooves on the aluminum alloy surface could not be filled completely, as shown in Figure 15a. This is because molten PA66 has a large viscosity and requires sufficient welding force to cause it to flow. When the welding force was optimal, no obvious defect could be seen in the joint (Figure 15b). When the welding force is too high, as shown in Figure 15c,d, there will be a large area of unfilled and unbonded metal surface in the joint because of the extrusion of molten CF/PA66. In addition, the extrusion of CF/PA66 also led to the thinning of the CF/PA66 sheet, resulting in a significant decrease in the peak load of the joint.

#### 3.2.3. Effect of Welding Time

**Figure 16:**
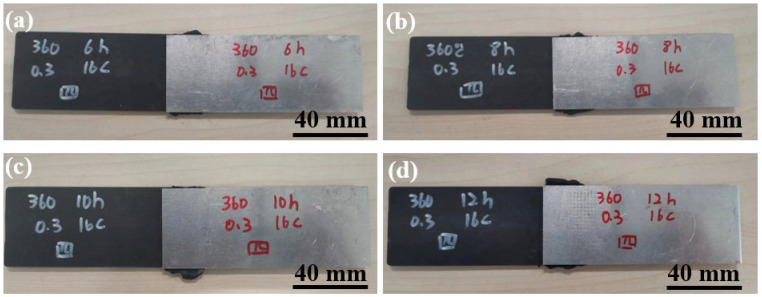
Macroscopic morphology of lap joints under four welding times: (**a**) 6 s; (**b**) 8 s; (**c**) 10 s; (**d**) 12 s.

Figure 16 shows the macroscopic morphologies of joints made with different welding times. No obvious difference can be observed among the four joints.

**Figure 17:**
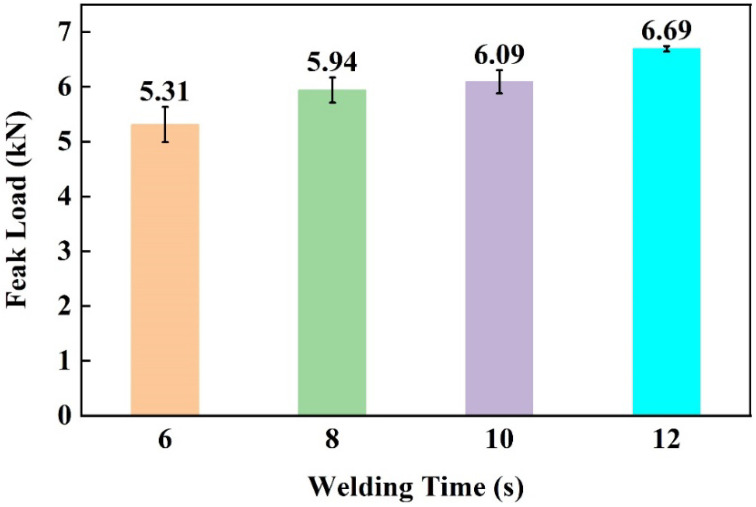
Effect of welding time on the peak load of joints.

**Figure 18:**
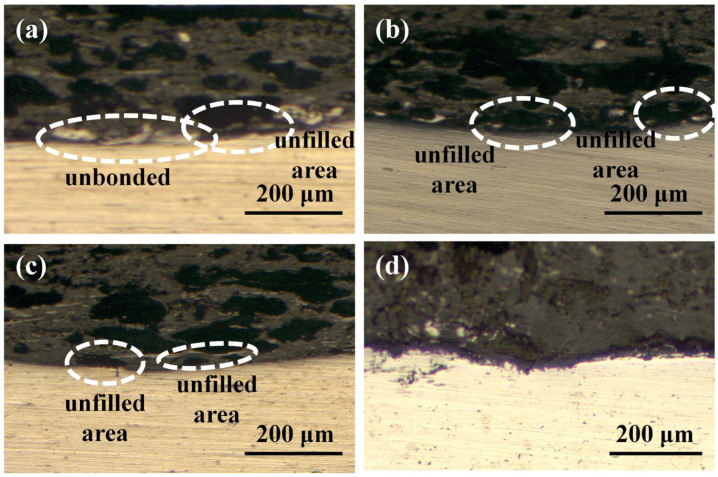
Cross-sectional morphologies of the joints made with different welding times: (**a**) 6 s; (**b**) 8 s; (**c**) 10 s; (**d**) 12 s.

Figure 17 shows the effect of welding time on the peak load of joints. It can be seen that the peak load increased as the welding time increased. This can be explained by the cross-sectional microstructures of Al/CFRTP joints, as shown in Figure 18. According to the cross-sections of the joints shown in Figure 18, some unbonded areas can be seen in the joints made with short welding time. This indicates that the wetting and bonding of CF/PA66 to the AA6061 requires sufficient time.

#### 3.2.4. Analysis of Fracture Morphology

**Figure 19:**
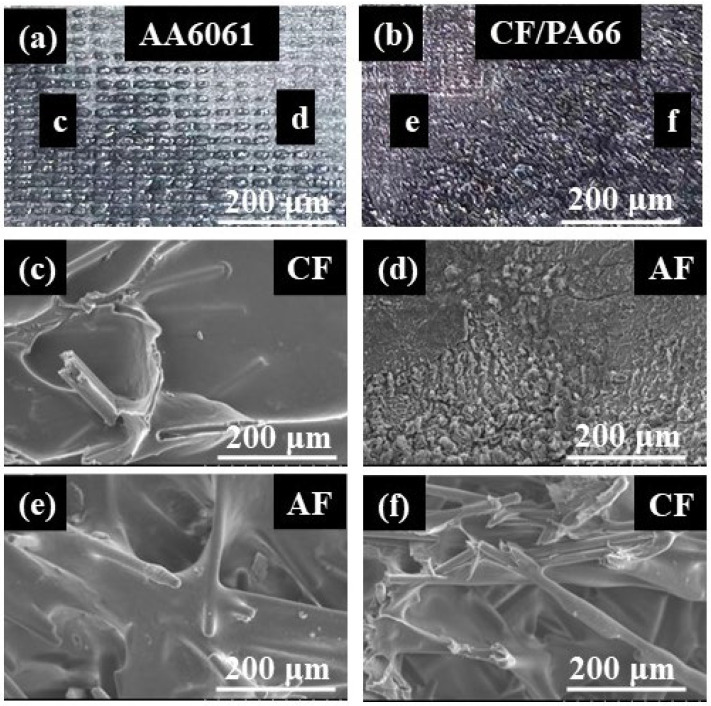
Fracture morphology of a joint with relatively low peak load: (**a**) fracture on the aluminum alloy side; (**b**) fracture on the CF/PA66 side; (**c**) area c on the aluminum alloy side; (**d**) area d on the aluminum alloy side; (**e**) area e on the CF/PA66 side; (**f**) area f on the CF/PA66 side (AF stands for adhesive failure; CF stands for cohesive failure).

When the joint is subjected to external loads, stress concentration will occur in the weak area at the interface, leading to the failure of the joint. Figure 19 shows the fracture surface morphology of a joint with a relatively low peak load (~4000 N) (welding time 6 s, temperature 320 °C, welding force 460 N, cooling time 10 s, No. 1 surface morphology). It can be seen that when the joint strength was low, the molten resin could not completely cover the surface of the aluminum alloy, and the processed grooves can still be seen, as shown in Figure 19a. Figure 19d shows a bare aluminum alloy surface. Its corresponding fracture position on the CF/PA66 side is shown in Figure 19e. It can be seen that some fibers were completely coated inside the resin. These phenomena indicate that this location experienced adhesive failure, i.e., interfacial bond failure between the CF/PA66 and the AA6061. In some locations, resin and fibers can also be observed on the AA6061 side, as shown in Figure 19c. Its corresponding fracture position on the CF/PA66 side is shown in Figure 19f. This failure is a cohesive failure since both sides were covered with a polymer matrix. Generally, cohesive failure is considered the preferred failure mode in a tensile–shear test because it means the bonding strength between the polymer and metal is stronger than the shear strength of the polymer matrix. Since only a small area of the welded joint experienced cohesive failure, leading to a relatively low peak load.

**Figure 20:**
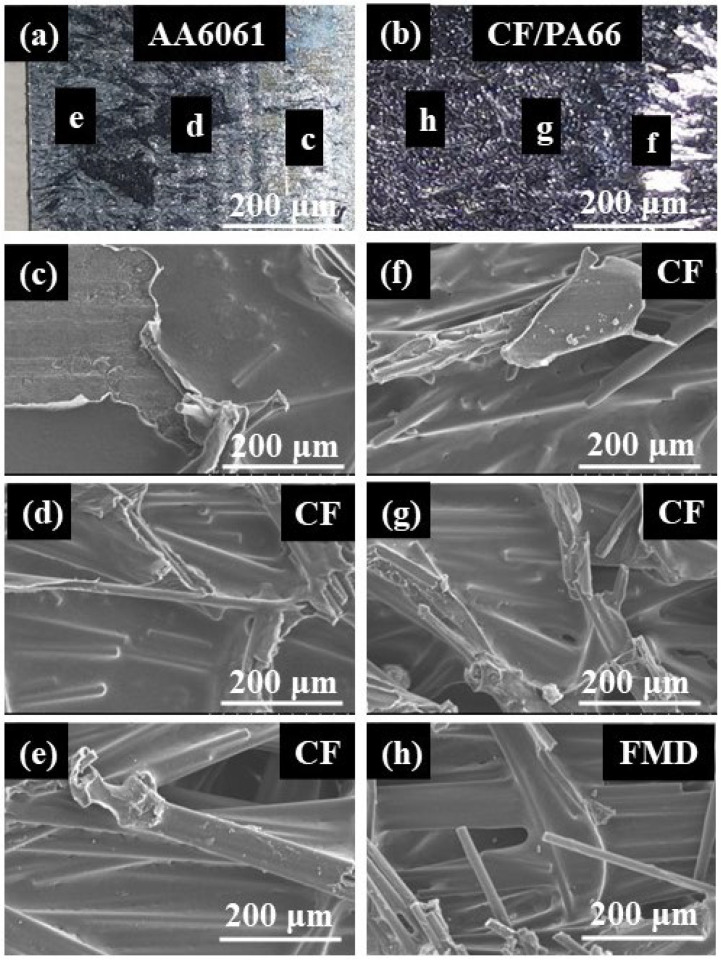
Fracture morphology of a joint with optimal peak load: (**a**) fracture on the aluminum alloy side; (**b**) fracture on the CF/PA66 side; (**c**) area c on the aluminum alloy side; (**d**) area d on the aluminum alloy side; (**e**) area e on the aluminum alloy side; (**f**) area f on the CF/PA66 side; (**g**) area g on the CF/PA66 side; (**h**) area h on the CF/PA66 side (CF stands for cohesive failure; FMD stands for fiber-matrix debonding).

The fracture morphology of a joint made with the optimal parameters is shown in Figure 20. It can be observed that the surface of the aluminum alloy was almost covered with CF/PA66. Only a small area of the joint experienced adhesive failure, as shown in Figure 20c. The aluminum alloy in most areas of the fracture (Figure 20d–g) was covered in resin, indicating cohesive failure had occurred. A small amount of bared fibers was observed in Figure 20h, indicating the occurrence of fiber-matrix debonding.

## 4. Conclusions

This study employed HPW to join AA6061 and CF/PA66. Jet-ECM was used to make grooves on the aluminum alloy surface. The process parameters (welding temperature, welding time, welding force, cooling time, and surface morphology) were optimized through orthogonal experiments. The main conclusions are given as follows.

(1) Aluminum alloy surface morphology had the greatest impact on the joint performance, followed by welding force, welding time, welding temperature, and cooling time. With a comparable total surface area of grooves, the grooves with a wider width and shallower depth contributed to higher joint strength.

(2) There existed an optimal welding force that maximized the joint strength. A longer welding time facilitated the wetting and bonding of the molten resin to the aluminum alloy, resulting in a stronger joint. Welding temperature had limited influence on the joint performance.

(3) Under the optimal parameters, the fracture morphology in the AA6061 side was almost covered with CF/PA66. Most of the joints experienced cohesive failure, and there was fiber-matrix debonding in a small area.

One limitation of this study is that the welding temperature used in the experiment was the temperature of the heated copper block, not the temperature of the welding interface, which may cause some deviations. Our next work will use more accurate test methods to study the impact of the welding interface temperature on joint performance. In addition, we will attempt to further improve the joint strength, such as further optimizing the surface morphology or using silane coupling agents to improve the bonding ability between metal and polymer.

## Figures and Tables

**Table 1:** Chemical composition of 6061 aluminum alloy (wt%).

Element	Al	Fe	Cu	Mn	Mg	Si	Zn	Ti	Al
AA6061	96	0.7	0.25	0.15	1.0	0.6	0.25	0.15	Bal.

**Table 2:** Properties of AA6061 and CF/PA66 sheets.

Material	Melting Point (°C)	Elastic Modulus (GPa)	Tensile Strength (MPa)	Elongation (%)
AA6061	650	68.7	311	12.5
CF/PA66	260	29.5	335	1.4

**Table 3:** Designed groove dimensions of the four surface morphologies of aluminum alloy sheet.

	No. 1	No. 2	No. 3	No. 4
Processing width (mm)	0.372	0.551	0.711	0.881
Processing depth (mm)	0.073	0.054	0.044	0.035
Spacing (mm)	0.372	0.551	0.711	0.881
Groove quantity in width (W) direction	46	32	25	20
Groove quantity in length (L) direction	19	14	11	9
Total surface area of grooves (mm^2^)	26.065	25.944	25.812	25.665

**Table 4:** Processing parameters for the four surface morphologies.

Surface Morphology	Nozzle Thickness (mm)	Electrolyte	Flow Rate (m/s)	Processing Time (s)	Processing Current (A)
No. 1	0.1	20%NaCl	4.28	10	1.8
No. 2	0.2	20%NaCl	4.28	10	1.8
No. 3	0.3	20%NaCl	4.28	10	1.8
No. 4	0.4	20%NaCl	4.28	10	1.8

**Table 5:** Five four-level factors orthogonal array test.

	Temperature (°C)	Welding Time (s)	Welding Force (N)	Cooling Time (s)	Surface Morphology
Level 1	320	6	460	10	No. 1
Level 2	340	8	720	12	No. 2
Level 3	360	10	980	14	No. 3
Level 4	380	12	1240	16	No. 4

The welding forces of 460, 720, 980, and 1240 N correspond to 0.2, 0.3, 0.4, and 0.5 MPa in the welding machine settings, respectively.

**Table 6:** Univariate experiments.

	Temperature (°C)	Welding Time (s)	Welding Force (N)	Cooling Time (s)	Surface Morphology
Group 1	360	12	720	16	Nos. 1, 2, 3, 4
Group 2	360	12	460, 720, 980, 1260	16	No. 4
Group 3	360	6, 8, 10, 12	720	16	No. 4

**Table 7:** Tensile–shear test results of the orthogonal array test.

No.	Temperature (°C)	Welding Time (s)	Welding Force (N)	Cooling Time (s)	Surface Morphology	Peak Load (N)			
						1	2	3	4
1	320	6	460	10	No. 1	4446.5	3555.8	3068.3	3681.5
2	320	8	720	12	No. 2	5415.1	4839.5	3718.7	5372.2
3	320	10	980	14	No. 3	5190.7	5668.5	4342.4	4025.5
4	320	12	1240	16	No. 4	5363.9	5963.8	4499.7	4732.3
5	340	6	720	14	No. 4	5541.1	5349.1	4281.3	5305.8
6	340	8	460	16	No. 3	4996.9	4890.1	5477.3	4363.1
7	340	10	1240	10	No. 2	3969.9	4253.9	4901.4	4152.9
8	340	12	980	12	No. 1	4455.7	4394.7	1508.8	4983.3
9	360	6	980	16	No. 2	5376.6	4052.1	3821.8	5417.5
10	360	8	1240	14	No. 1	4083.1	4118.8	3862.3	3963.6
11	360	10	460	12	No. 4	5381.3	5328.1	5131.8	4872.3
12	360	12	720	10	No. 3	6663.9	6343.9	5322.2	4899.1
13	380	6	1240	12	No. 3	3550.1	3860.2	5558.6	4058.8
14	380	8	980	10	No. 4	4726.9	4563.6	5198.6	4412.5
15	380	10	720	16	No. 1	4618.7	4819.9	3788.1	4546.9
16	380	12	460	14	No. 2	6088.5	6379.1	4176.9	3542.8

**Table 8:** Range analysis of the orthogonal array test.

K*_i_*	Temperature	Welding Time	Welding Force	Cooling Time	Surface Morphology
K_1_	18,470.8 N	17,730.8 N	18,844.8 N	18,540.4 N	16,074.0 N
K_2_	18,306.0 N	18,500.4 N	20,206.4 N	18,207.2 N	18,869.6 N
K_3_	19,656.4 N	18,748.0 N	18,134.8 N	18,980.0 N	19,803.2 N
K_4_	18,472.4 N	19,929.6 N	17,723.2 N	19,182.4 N	20,163.2 N
R	1350.4 N	2198.8 N	2483.2 N	642.0 N	4089.2 N
Degree of effect	surface morphology > welding force > welding time > welding temperature > cooling time				
Optimal level	360 °C	12 s	720 N	16 s	No. 4
Optimal process parameters	No. 4 surface morphology, welding force 720 N, welding time 12 s, welding temperature 360 °C and cooling time 16 s				

## Data Availability

The data presented in this study are available upon request from the corresponding author.
